# Whole-Genome Metagenomic Analysis of Functional Profiles in the Fecal Microbiome of Farmed Sows with Different Reproductive Performances

**DOI:** 10.3390/microorganisms12112180

**Published:** 2024-10-29

**Authors:** Hiroto Miura, Takamitsu Tsukahara, Ryo Inoue

**Affiliations:** 1 Laboratory of Animal Science, Department of Applied Biological Sciences, Faculty of Agriculture, Setsunan University, Hirakata 573-0101, Japan; hiroto.miura@setsunan.ac.jp; 2 Kyoto Institute of Nutrition & Pathology, Kyoto 610-0231, Japan; tsukahara@kyoto-inp.co.jp

**Keywords:** pig, reproductive performance, gut microbiota, CAZyme, fiber degradation, short-chain fatty acid

## Abstract

Recent studies suggested an association between the reproductive performance of sows and their gut microbiota. To understand how the gut microbiota affect the reproductive performances of sows, we conducted a whole-genome metagenomic analysis on the fecal microbial functional profiles of sows with high and low reproductive performances. We used 60 sows from six farms (10 sows/farm), including 30 sows from three farms with higher reproductive performances (the mean number of weaned piglets/sow/year) (group H) and 30 sows from three farms with lower performances (group L). Fecal microbial DNA was subjected to a whole-genome metagenomic analysis. Biomarker exploration analysis identified “carbohydrate transport and metabolism” as the most discriminative function enriched in group H. Further analysis of carbohydrate-active enzymes revealed that the fecal microbiome of group H had a greater capacity to degrade dietary fiber, specifically cellulose and pectin. Group H also exhibited higher fecal short-chain fatty acid (SCFA) concentrations than group L, with the abundances of cellulose- and pectin-degrading genes showing significant positive correlations with fecal SCFA concentrations. Taxonomic analysis indicated greater contributions of *Prevotella*, *Treponema*, *Ruminococcus*, and *Fibrobacter* to cellulose and pectin degradation in the fecal microbiome in group H. In conclusion, higher reproductive performances of sows were, at least in part, associated with a greater microbial capacity for degrading cellulose and pectin, resulting in a higher SCFA production in the hindgut.

## 1. Introduction

Pigs are an important livestock species as they are a vital source of dietary animal protein and medical resources. Presently, estimated as the total carcass weight, the world’s pig meat production is about 121.7 million tons, which accounts for one-third of the total livestock meat production [1]. As the world population grows, global pork demand is expected to further reach up to 207 million tons by 2050 [2]. In addition, pigs have recently been considered the most promising donor animals in xenotransplant medicine [3]. These facts highlight the need to improve the stability and efficiency of pig production.

Among the productive traits of pigs, the reproductive performance of sows is a crucial aspect critically affecting farm productivity [4]. Recently, an association between the reproductive performance of sows and their gut microbiota has been suggested. Indeed, studies have reported differences in the fecal microbial compositions between sows with high and low reproductive performances within a farm [5,6,7]. In addition, our previous large inter-farm study confirmed that farmed sows with high reproductive performances, namely a high number of weaned piglets per sows per year, had different fecal microbial compositions from farmed sows with low performances [8]. These findings indicate that the sow’s reproductive performance can be enhanced by achieving a specific gut microbiota community structure. However, the mechanisms by which the gut microbiota community structures affect the sow’s reproductive performance remain unclear.

There are many possible mechanisms by which the animal gut microbiota can influence the host phenotype, including the effects on the nutritional, physiological, immunological, and neurological status of the host [9,10]. To understand how the gut microbiota affect the reproductive performances of sows, it is essential to focus not only on the taxonomic profile of the gut microbiota but also on its functional aspects, i.e., what types and numbers of functional genes are present in the gut microbiota [11,12]. Based on this context, whole-genome metagenomic sequencing (WG metagenomic sequencing), which analyzes both the taxonomy and the functions of microbial communities, seems to be a reasonable approach to study the association between the gut microbiota and the host phenotypes. In fact, in studies using dairy ruminants, WG metagenomic analyses showed that differences in the rumen microbial capacity for amino acid and fatty acid biosynthesis influence the host’s milk production traits [13,14]. Similarly, a WG metagenomic analysis on pigs showed that the gut microbial capacity for amino acid metabolism and biosynthesis was associated with the host’s feed efficiency traits [15]. These studies highlight the advantage of WG metagenomic analysis in interpreting the association between gut microbiota and sows’ reproductive performances and their mode of action.

In the present study, we conducted a WG metagenomic analysis on the fecal microbial functional profiles of sows with high and low reproductive performances, which had not been previously explored. Here, we hypothesize that sows with different reproductive performance would have different gut microbiota, not only taxonomically, but also functionally. The aim of this study was to examine this hypothesis and, if supported, to further address the following questions: which gut microbiota functions are important for sow reproductive performance and which microbial taxa are responsible for these functions? In addition, this study employed an in vitro batch culture experiment which helped to further validate the phenotypic importance of key microbial functions identified by WG metagenomic analysis.

## 2. Materials and Methods

### 2.1. Experiment 1: Whole-Genome Metagenomic Analysis of Functional Profiles in the Fecal Microbiome of Sows with High and Low Reproductive Performances

#### 2.1.1. Study Design and Samples

In our previous study [8], a total of 180 sows from 18 farms (10 sows/farm) were used to investigate the fecal microbial taxonomic composition. Briefly, these 18 farms were selected as per breeding lines (L × W or W × L) and reproductive performance (the mean number of weaned piglets/sow/year). As a result, nine farms from the top quartile and nine from the bottom quartile of the reproductive performance range were selected. All sows used were healthy and in the mid-gestation period, with parities between 2 and 5 (equivalent to 19–36 months of ages) and body condition scores [16] between 3 and 3.5. To further compare the functional profiles in the fecal microbiome of sows showing different reproductive performances, we conducted a WG metagenomic analysis on 60 sows from six farms (10 sows/farm). As a result, three farms with the highest performances in the above-mentioned nine top-quartile farms (denoted as group H, *n* = 30) and three farms with the lowest performances in the above-mentioned nine bottom-quartile farms (denoted as group L, *n* = 30) were selected. Sows in all selected farms were given a commercial, complete feed mixture which was formulated to meet their nutrient requirements. The reproductive performances of groups H and L are shown in Table S1. We subjected the same DNA used in a previous study [8] to a WG metagenomic analysis for the current work. All experimental procedures were followed in accordance with the guidelines issued by the Ministry of Education, Culture, Sports, Science and Technology of Japan and the Science Council of Japan. As described in a previous study [8], the experiments were exempted from ethical evaluations because all animals used were commercially raised and reared in conventional swine farms under the supervision of the local veterinary and the samples were collected on site.

#### 2.1.2. Whole-Genome Metagenomic Sequencing, Quality Control of Reads, and Assembly

WG metagenomic libraries were constructed from 200 ng of fecal DNA using a NEBNext Ultra II FS DNA Library Prep Kit for Illumina^®^ (New England Biolabs, Ipswich, MA, USA) as per the manufacturer’s protocol, and we sequenced them on Illumina HiSeq 1000 (paired-end, 2 × 150 bp) and NovaSeq X platforms (paired-end, 2 × 150 bp). All experiments were conducted under controlled temperature (23 ± 5 °C) and humidity (55 ± 10% RH) conditions. The quality of the constructed libraries was checked at each point described in the protocol. Furthermore, the sequencing company verified the quality of the library before sequencing. As for consumables, unless otherwise stated, we used standard plastic consumables available from Watson (Kobe, Japan) and Corning (Corning, NY, USA). All the sequence data were deposited in the DDBJ Sequence Read Archive under the BioProject accession number PRJDB18770. Using fastp software v.0.22.0 [17], raw reads were filtered to remove adapters, low-quality bases (quality scores < 20), and short reads (<50 bp). The resulting reads were aligned to the pig reference genome (*Sus scrofa* v.11.1, NCBI accession no. GCF_000003025) [18] using Bowtie2 v.2.4.1 [19] to filter out the host’s DNA contamination. The remaining reads were referred to as the filtered reads. The filtered reads were assembled individually using metaSPAdes v.3.15.5 [20] and co-assembled using MEGAHIT v.1.2.9 [21]. The resulting contigs were pooled and those with a length ≥ 500 bp were retained for downstream analyses.

#### 2.1.3. Construction of Non-Redundant Gene Catalog and Annotation

Open reading frames (ORFs) of assembled contigs were predicted using Prodigal v.2.6.3 [22] with the “meta” option. The predicted ORF sequences with a length ≥ 150 bp were retained and clustered with a cutoff of >95% identity and >90% coverage using CD-HIT v.4.8.1 [23]. The longest sequence in each cluster was used as a representative sequence of the non-redundant (NR) gene catalog.

Taxonomic annotation of the NR gene catalog was carried out using Kaiju v.1.6.2 [24] in “greedy” mode with default settings against the “nr_euk” database (NCBI nr database with microbial eukaryote data) ver.2023-05-10. Functional annotation of the NR gene catalog was obtained using eggNOG-Mapper v.2.1.12 [25] with the default settings against the eggNOG database v.5.0 [26]. Functional abundance profiles were summarized based on eggNOG-ID and the COG functional category. Furthermore, the NR genes encoding carbohydrate-active enzymes (CAZymes), including glycoside hydrases (GHs), glucosyl transferases (GTs), polysaccharide lyases (PLs), carbohydrate esterases (CEs), carbohydrate binding modules (CBMs), and auxiliary activities (AAs), were predicted using the local version of dbCAN2 v.4.0.0 [27] and HMMER v.3.3.2, with the default settings. Relative abundances of genes were calculated as transcripts per million (TPM) using CoverM v.0.6.1 (https://github.com/wwood/CoverM, accessed on 23 July 2024), based on the fact that the original filtered reads of each sample were mapped back to the constructed NR gene catalog using Bowtie2. CAZyme-family genes with an average relative abundance (TPM) >1 and with a prevalence >20%, as well as microbial genera with an average relative abundance (% of total bacteria) >0.1% and with a prevalence >20%, were considered and used for analysis.

### 2.2. Experiment 2: In Vitro Batch Culture of Sow Feces with Cellulose and Pectin

The WG metagenomic analyses in Experiment 1 suggested that the gut microbiota of sows in group H had a higher capacity for dietary cellulose and pectin degradation than those in group L. To further evaluate the relationship between those microbial characteristics and the production of short chain fatty acids (SCFAs), an in vitro batch culture was conducted in Experiment 2. Based on a preliminary evaluation of functional profiles of the gut microbiota, we selected two farms out of six (which were not included in the Experiment 1) as the farms with sows with the highest and the lowest potential for fiber degradation (Supplementary Figure S1). Feces were sampled from five sows (2nd–5th parity) from each of the two farms and the samples from the same farm were pooled at an equivalent volume. These pooled feces were diluted with 19 volumes of an anaerobic sodium phosphate buffer (50 mmol/L, pH 6.6) and then used as inoculum. In total, 20 mL of inoculum was dispensed into a 30 mL glass bottle. Either 100 mg of cellulose (microcrystalline cellulose Avicell; Merck, Darmstadt, Deutschland) or pectin (citrus-derived pectin; FUJIFILM Wako pure chemical corporation, Osaka, Japan) was added as a substrate to the above bottles in triplicates, as technical replicates. As control, bottles with no substrate were prepared in triplicates. The headspace of each bottle was flushed with N_2_ gas and sealed with a butyl rubber stopper and a plastic screw cap, and the bottles were incubated with gentle shaking at 38 °C for 24 h. After incubation, the cultures were subjected to SCFA measurement as per Tsukahara et al. [28].

### 2.3. Statistical Analyses

All statistical tests were conducted using R v.4.3.1 [29]. The Shapiro–Wilk test confirmed that some of the gene abundance in the present dataset does not follow a normal distribution. As a result, we applied non-parametric statistical methods throughout our analyses, as follows. Differences in the composition of functional genes in the fecal microbiome of groups H and L were analyzed by a principal coordinate analysis (PCoA) with Bray–Curtis dissimilarity matrices using vegan packages v.2.5.6 [30]. Statistical significances of the PCoA were estimated using a permutational multivariate analysis of variance (PERMANOVA) with 9999 permutations. Values were considered statistically significant if *p* < 0.05. Biomarker COG-category functions and CAZyme-family genes enriched in groups H and L were determined by using a linear discriminant analysis (LDA) coupled with the effect size (LefSe) algorithm [31]. For the LEfSe analysis, the statistical significance was set at an LDA score > 2.5, with *p*-values < 0.05 and a false discovery rate (FDR) < 0.15. The relative abundances of selected CAZyme genes and bacterial genera were compared between groups using the Wilcoxon rank-sum test, and *p*-values < 0.05 with FDR < 0.15 were considered statistically significant.

## 3. Results

### 3.1. Summary of the Whole-Genome Metagenomic Analysis

The WG metagenomic sequencing yielded a total of 947 million high-quality reads, with 15.8 ± 1.1 million reads (the mean ± the standard error) per sample. A summary of the constructed gene catalog is presented in Supplementary Table S2. Briefly, we obtained a total of 7,477,793 non-redundant (NR) genes (the N50 length of 717 bp). Among the NR genes, 5,129,426 (68.5%) genes and 130,516 (1.7%) genes were annotated to the COG functional categories and CAZyme genes, respectively. The former and latter genes consisted of 82.4% and 93.7% of bacteria, respectively. Thus, the downstream taxonomic analysis was focused only on bacteria.

### 3.2. Comparison of Functional Profiles of the Fecal Microbiome of Sows Between Groups H and L

To investigate the broad range of the functional profiles of the sows’ fecal microbiome, we first summarized the gene abundances based on the COG functional category (Table S3). The results showed that eight categories of known functions had average abundances >5.0% of the total annotated genes, including categories L (replication, recombination and repair), M (cell wall/membrane/envelope biogenesis), K (transcription), J (translation, ribosomal structure and biogenesis), E (amino acid transport and metabolism), G (carbohydrate transport and metabolism), C (energy production and conversion), and P (inorganic ion transport and metabolism).

**Figure 1:**
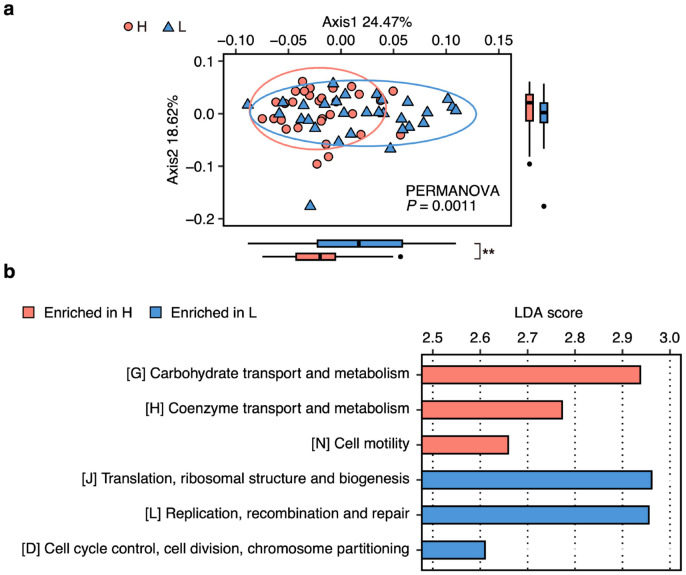
Comparison of broad range of functional profiles in the fecal microbiome of sows in groups H and L. (**a**) Principal coordinate analysis on Bray–Curtis dissimilarities based on the relative abundances of functional genes annotated by eggNOG-mapper. Individual symbols represent individual samples (red circle, sows H; blue triangle, sows L). Significance was tested using a permutational multivariate analysis of variance (PERMANOVA, permutations = 9999). Boxplots show distributions of samples in groups H (red) and L (blue) along PC1 and PC2 (** *p* < 0.01, Wilcoxon rank-sum test). (**b**) Differentially abundant functions in fecal microbiome of sows in groups H and L. Relative abundances of functional genes were summarized based on COG functional categories and compared between groups using a linear discriminant analysis of effect size (LEfSe). Only categories with significance (LDA score > 2.5, *p* < 0.05, FDR < 0.15) are shown.

The PCoA analysis suggested significant differences in compositions of the overall functional genes in the fecal microbiome between groups H and L (*p* = 0.001; Figure 1a). The LEfSe analysis revealed that the relative abundance of genes belonging to category G (carbohydrate transport and metabolism) was more enriched in group H than in group L, followed by genes belonging to category H (coenzyme transport and metabolism) and category N (cell motility) (LDA > 2.5, *p* < 0.05, FDR < 0.15; Figure 1b). In contrast, genes belonging to categories J (translation, ribosomal structure, and biogenesis), L (replication, recombination, and repair), and D (cell cycle control, cell division, chromosome partitioning) were enriched only in group L (LDA > 2.5, *p* < 0.05, FDR < 0.15; Figure 1b).

### 3.3. Comparison of the CAZyme Gene Profile and Its Taxonomic Affiliations with the Fecal Microbiome of Sows Between Groups H and L

The functional profile analysis suggested that the fecal microbiome of sows in group H had a significantly higher capacity for carbohydrate metabolism than those in group L (Figure 1). Therefore, to further explore the detailed carbohydrate-metabolism function being linked to the sows’ reproductive performance, we analyzed the profile of genes encoding CAZymes.

**Figure 2:**
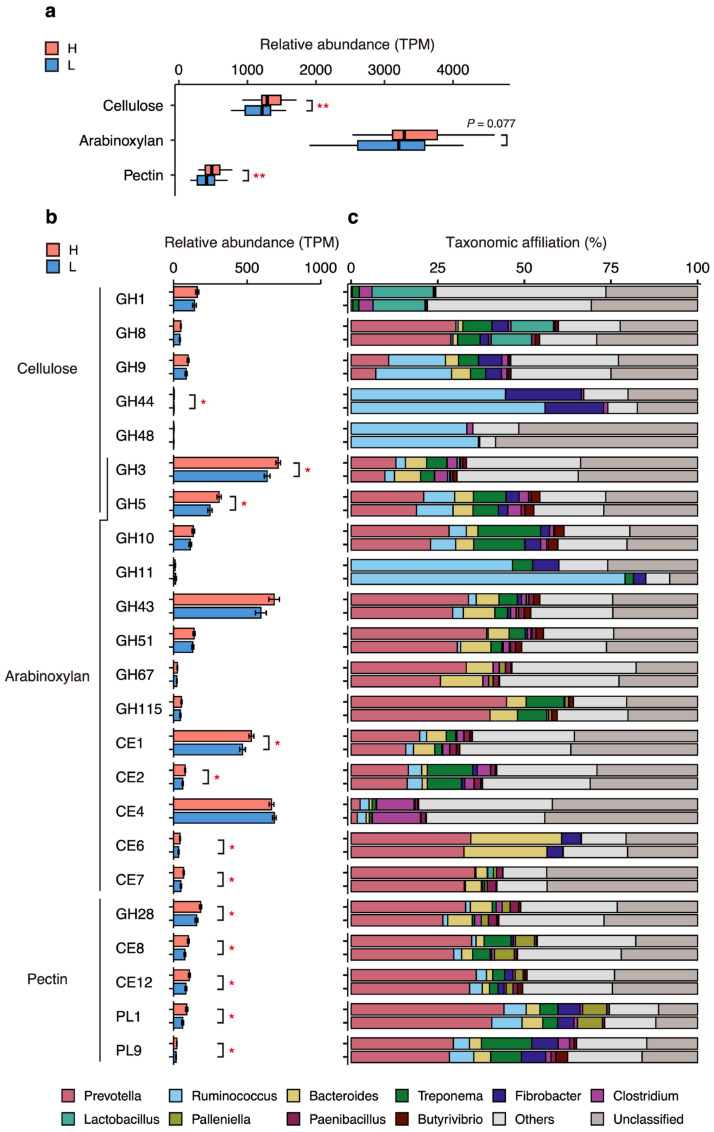
Comparison of relative abundances of CAZyme genes involved in dietary fiber degradation and their taxonomic affiliations. (**a**) Cumulative relative abundances of cellulose-, arabinoxylan-, and pectin-degrading genes. Targeted CAZyme families were selected as per Flint et al. [34] (cellulose, GH1, GH3, GH5, GH8, GH9, GH44, and GH48; arabinoxylan, GH3, GH5, GH10, GH11, GH43, GH51, GH67, GH115, CE1, CE2, CE4, CE6, and CE7; pectin, GH28, CE8, CE12, PL1 and PL9). Significance was tested using the Wilcoxon rank-sum test (** *p* < 0.01). (**b**) Relative abundance of individual CAZyme family genes targeting cellulose, arabinoxylan, and pectin. Bars and error bars represent the averages and the standard errors, respectively. Significance was tested using the Wilcoxon rank-sum test (* *p* < 0.05, FDR < 0.15). (**c**) The taxonomic affiliation of selected CAZyme family genes at the genus level. Proportions (%) of taxonomic affiliations were calculated by dividing the TPM assigned to each bacterial genus in each CAZyme family gene by the total TPM for each CAZyme family gene. Only bacterial genera showing >5% proportion in at least one CAZyme family in either group H or L are shown as major fiber degraders.

The CAZyme analysis detected genes belonging to a total of 254 CAZyme families in the fecal microbiome, including 117 glycoside hydrases (GHs), 51 glucosyl transferases (GTs), 45 carbohydrate binding modules (CBMs), 19 polysaccharide lyases (PLs), 17 carbohydrate esterases (CEs), and 5 auxiliary activities (AAs) (Supplementary Table S4). We found that the fecal microbiome of group H had significantly higher relative abundances of genes encoding GHs, GTs, CEs, and PLs (*p* < 0.05; Supplementary Figure S2a) than those of group L, and that the CAZyme gene compositions were significantly different between groups H and L (*p* = 0.003; Supplementary Figure S2b). Notably, the LEfSe analysis showed that twelve CAZyme family genes were specifically enriched in group H, nine of which were involved in the degradation of plant carbohydrates, such as cellulose, hemicellulose, pectin, and starch (LDA score > 2.5, *p* < 0.05, FDR < 0.15; Supplementary Figure S2c). Therefore, we further compared the abundances of genes targeting typical fibers in swine diets between groups and analyzed their taxonomic affiliations. Here, we focused on cellulose, arabinoxylan, and pectin as the main dietary fibers for pigs [32,33] and selected the CAZyme families that degrade these fibers, according to Flint et al. [34]. The cumulative relative abundances of cellulose- and pectin-targeting genes were significantly higher (*p* < 0.01) in group H than in group L. Arabinoxylan-degrading genes were non-statistically higher (*p* = 0.077) in group H when compared with those in group L (Figure 2a). For the individual CAZyme family genes (Figure 2b), the fecal microbiota in group H showed significantly higher abundances of GH3 (beta-glucosidase/beta-xylosidase), GH5 (endo-cellulase/endo-xylanase), GH28 (endo-polygalacturonase), GH44 (endo-cellulase), CE1 (acetyl xylan esterase/feruloyl esterase), CE2 (acetyl xylan esterase), CE6 (acetyl xylan esterase), CE7 (acetyl xylan esterase), CE8 (pectin metyl esterase), CE12 (pectin acetyl esterase), PL1 (pectin/pectate lyases) and PL9 (pectin/pectate lyases) (*p* < 0.05, FDR < 0.15) than those in group L. Furthermore, group L showed no significantly enriched CAZyme family.

For the taxonomic affiliation analysis, an average of 74% of genes in each selected CAZyme family was successfully classified at the genus level (Figure 2c). A total of 10 bacterial genera were identified as major fiber degraders with >5% affiliation to at least one CAZyme family in either group H or L, and they covered an average of 54% of genes in each selected family. Of these 10 bacterial genera, genes from *Prevotella*, *Ruminococcus*, *Bacteroides*, *Treponema*, and *Fibrobacter* were widely and predominantly distributed to the selected families, accounting for an average of 44% of genes in each selected family. Moreover, *Prevotella*, *Bacteroides*, and *Treponema* were predominant in most of the families. *Ruminococus* showed a more specific and dominant affiliation in GH9, GH11, GH44, and GH48. Similarly, and in contrast with other families, *Fibrobacter* preferentially possessed the genes of endo-acting CAZyme families including GH5, GH8, GH9, GH44, PL1, and PL9.

### 3.4. In Vitro Batch Culture Evaluation of the Relationship Between Fecal Microbial Capacity for Fiber Degradation and SCFA Production

**Figure 3:**
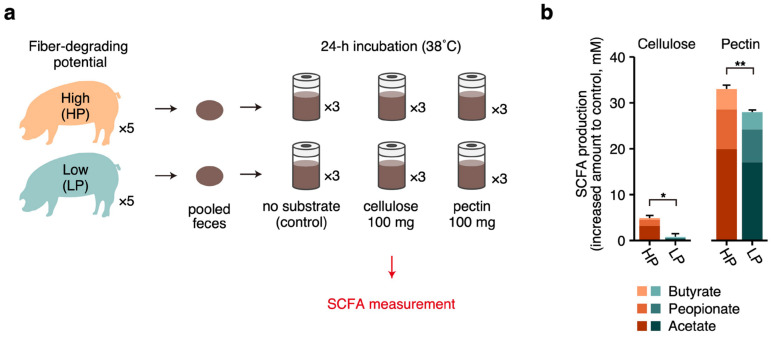
In vitro batch culture experiment to evaluate the relationship between the fecal microbial capacity for dietary fiber degradation and the fecal SCFA concentrations. (**a**) Summary of the in vitro batch culture experiment using feces derived from sows with high (HP) and low potential (LP) for dietary fiber degradation. (**b**) SCFA production after 24 h incubation of HP and LP feces with cellulose or pectin as substrates. Values represent increased amounts of SCFA concentrations compared to those of control, meaning net SCFA production. Bars and error bars represent the averages and the standard errors of total SCFA production. Significance was tested using Welch’s *t*-test (** *p* < 0.01, * *p* < 0.05).

To validate that a higher microbial capacity for cellulose/pectin degradation leads to a higher SCFA production, an in vitro batch culture was conducted in Experiment 2. We used feces sampled from sows with high (HP) and low potential (LP) for fiber degradation as inocula and incubated them with either cellulose or pectin (Figure 3a). After a 24 h incubation, the SCFA production was significantly higher in HP feces when compared with LP feces, with 6-fold higher SCFA production when cellulose was used as the substrate (Figure 3b).

## 4. Discussion

### 4.1. Differences in Functional Profiles of the Fecal Microbiome Among Sows with Different Reproductive Performance

Studies using the 16S rRNA gene sequencing approach have suggested that specific gut microbiota community structures are associated with higher reproductive performances of sows [5,6,7,8]. However, the understanding of the relationship between the gut microbiota and the host phenotype is difficult when only the taxonomic information is available [11,12]. Therefore, in the present study, we analyzed the fecal microbial functional profiles of sows with high and low reproductive performances. Based on the hypothesis that sows with different reproductive performances would have different gut microbial functional profiles, we aimed to identify the functions of the gut microbiota that are important for sow reproductive performance and the microbial taxa responsible for these functions.

We first comprehensively investigated the functional profiles in the fecal microbiome of sows in groups H and L. Among the COG categories, “carbohydrate transport and metabolism” was suggested as the most discriminative function enriched in group H. Further detailed CAZyme analysis revealed that the fecal microbiome of sows in group H had a greater capacity to degrade dietary fiber, specifically cellulose and pectin. Gut bacteria utilize carbohydrates derived from undigested feed that reach the hindgut, mainly dietary fiber, as an energy source and eventually metabolize them to SCFAs and gases [35]. SCFAs not only provide up to 25% of the host’s energy [36] but also regulate various physiological functions of the host such as gut motility, inflammatory response, and stress alleviation [35,37]. In addition, SCFAs produced in the gut microbiota have been reported to be involved in mediating reproductive function in sows, humans, and mice [38,39]. Therefore, our present results indicated that the gut microbial capacity to degrade cellulose and pectin could be one of the most important factors affecting the reproductive performances of sows via the production of SCFAs in the gut. Indeed, we observed that group H exhibited higher fecal SCFA concentrations than group L and the abundances of cellulose- and pectin-degrading genes showed significant positive correlations with fecal SCFA concentrations (Supplementary Figure S3). In addition, with an in vitro batch culture, we further validated that the gut microbiota of sows with a higher capacity for cellulose and pectin degradation could produce more SCFAs from same amounts of cellulose and pectin than those with a lower capacity. Interestingly, we also analyzed the distribution of genes related to SCFA biosynthesis (i.e., genes involved in the conversion of sugar into SCFAs) and found that their abundances were not significantly different between groups H and L (Miura et al., unpublished data). This may suggest that the gut microbial capacity for fiber degradation (i.e., the upstream function that decomposes dietary structural carbohydrates into sugars), not SCFA biosynthesis (i.e., the downstream function that converts sugars into SCFAs), is a more important function affecting gut SCFA production and, consequently, the reproductive performances of sows.

According to a review by Navarro et al. [40], cellulose, arabinoxylan, and pectin account for 5–20%, 10–65%, and 1–10%, respectively, of the total dietary fiber in major cereal grains. In agreement with those fiber abundances, arabinoxylan-degrading genes were the most abundant (Figure 2a) and the more widely distributed genes among various bacteria in both groups (Supplementary Figure S4). Therefore, the gut microbial capacity for arabinoxylan degradation may be more stable in sows than that for cellulose and pectin. This is because, in the present work, the capacity for arabinoxylan degradation was found to be less impactful on the SCFA concentrations. Therefore, hereinafter, we focused on the links between the sows’ reproductive performance and microbial capacity for cellulose and pectin degradation and not for arabinoxylan degradation.

### 4.2. Exploration of Key CAZyme Genes and Their Taxonomic Affiliations Linked to Sows’ Reproductive Performance

The distribution of individual CAZyme family genes provided a further insight into what types of enzymes are especially important for cellulose and pectin degradation in the hindgut of sows with high reproductive performances. Group H showed an enrichment of genes encoding GH3 and GH5, which are two of the most predominant beta-glucosidases and endo-cellulases in the rumen, respectively [41]. Endo-cellulases are essential for the generation of new chain ends and oligosaccharides, and beta-glucosidases further digest these products [42]. Our results suggested that the synergistic action of GH3 and GH5 is a key factor affecting the efficiency of cellulose digestion in the sows’ hindguts. Indeed, under the same feeding condition, pigs with higher fiber digestibility had higher abundances of GH3 and GH5 genes in the fecal microbiome than those with a lower digestibility [43]. In addition, enrichment of GH44 was observed in group H. In a rumen study, GH44 genes were shown to be relatively less abundant but highly expressed as mRNA [41]. Furthermore, the mRNA of GH44 was reported to be present more in the solid fraction of rumen content (i.e., feed fiber particle) than in the liquid fraction [44]. These reports seem to suggest that there may also be a special niche for GH44 in cellulose digestion in the sows’ hindgut. For the pectin degradation, all selected genes encoding debranching enzymes (CE8 and CE12) and endo-pectinase (GH28, PL1, and PL9) were enriched in group H. Debranching of side chains is the first step in microbial pectin degradation and increases the work of endo-pectinases [41,45]. Our results suggested that the synergistic work of different enzymes is also important for pectin degradation, similar to that described for cellulose digestion.

Next, we analyzed the taxonomic affiliations of CAZyme genes (Figure 2c). When we focused on the above-mentioned CAZyme families targeting cellulose (i.e., GH3, GH5, and GH44) and pectin (GH28, CE8, CE12, PL1, and PL9), greater contributions of *Prevotella*, *Bacteroides*, *Treponema*, *Ruminococcus*, and *Fibrobacter* were observed. Notably, except for GH44, in most of the focused families, *Prevotella* showed the highest proportion among the classified genera, suggesting its key roles in both cellulose and pectin degradation. In particular, all selected pectin-degrading genes were dominated by *Prevotella*, indicating that *Prevotella* could independently break down and use heteropolysaccharide pectin. Many, but not all, correlative studies using 16S rRNA gene sequencing have shown a positive relationship between the abundance of swine gut *Prevotella* and improved host production outcomes [46,47,48,49]. Our WG metagenomic analysis indicated that part of the mechanism behind this relationship may be explained by the greater ability of *Prevotella* to utilize various carbohydrates, leading to increased SCFA production. Of the selected families, GH44 (endo-cellulase) showed a unique taxonomic affiliation pattern with *Ruminococcus* and *Fibrobacter*, occupying prominent proportions. Both genera are known as the most pivotal cellulolytic rumen bacteria inhabiting the fiber particles of rumen content [50,51]. The results in the present work also seem to emphasize a special niche of these bacteria and their GH44 genes for cellulose digestion in the pig hindgut and, consequently, for SCFA production and the enhancement of the host’s reproductive performance. In the present study, of the above-mentioned five bacteria, *Prevotella* and *Treponema* exhibited significantly higher abundances (*p* < 0.01) in group H when compared with those in group L (Supplementary Table S5). In addition, although incorporating three farms each in groups H and L failed to show significance, our previous surveillance incorporating nine farms into each experimental group showed significantly higher abundances of *Ruminococcus* and *Fibrobacter* in farms with higher reproductive performances [8]. Hence, we speculated that these four taxa (*Prevotella*, *Treponema*, *Ruminococcus*, and *Fibrobacter*) are the key taxa for greater capacity for cellulose and pectin degradation in group H.

We acknowledge that this study had some limitations due to it being conducted on commercial farms: to facilitate farm cooperation, we only requested healthy sows with parities between 2 and 5 (equivalent to 19–36 months of age) and body condition scores between 3 and 3.5 and who were in the mid-gestation period, meaning that detailed individual data were not available. Similarly, data on body weight were not available, as this is not typically measured in Japanese commercial farms. As a result, this may constrain the study’s ability to fully investigate the relationship between gut microbial capacity for fiber degradation and host physiological parameters beyond reproductive performance. Furthermore, this study focused only on the major fibers in the swine diet, excluding less prevalent fibers. To confirm and expand the present findings, future research with larger sample sizes, incorporating more farms and more detailed individual data, is needed.

## 5. Conclusions

In conclusion, the present study revealed that higher reproductive performances of sows were, at least in part, associated with a greater microbial capacity for degrading cellulose and pectin, resulting in higher SCFA production in the hindgut. We also showed that those gut microbial functions were mainly driven by four key taxa, namely *Prevotella*, *Treponema*, *Ruminococcus*, and *Fibrobacter*.

## Data Availability

Raw sequences have been deposited in the DDBJ Sequence Read Archive under the accession no. PRJDB18770.

## Supplementary Materials

The following supporting information can be downloaded at https://www.mdpi.com/article/10.3390/microorganisms12112180/s1: Table S1: Parameters of reproductive performance in farms H and L; Table S2: Summary of sequencing and constructed gene catalog; Table S3: Relative abundance (transcripts per million, TPM) of genes assigned to COG functional categories; Table S4: Relative abundances of detected CAZyme family genes in the fecal microbiomes of groups H and L; Table S5: Relative abundances of genus-level bacterial taxa in the fecal microbiomes of groups H and L; Figure S1: Evaluation of the fiber-degrading potential of the sows’ gut microbiota in six farms; Figure S2: Comparison of the carbohydrate-active enzyme (CAZyme) gene profiles in the fecal microbiome of sows in groups H and L; Figure S3: Fecal SCFA production and its relation to fecal microbial capacity for dietary fiber degradation in sows; Figure S4: Total number of bacterial genera detected in affiliation with cellulose-, arabinoxylan-, and pectin-degrading genes.
