# Supplementary material for: Whole-Genome Metagenomic Analysis of Functional Profiles in the Fecal Microbiome of Farmed Sows with Different Reproductive Performances
Source: Microorganisms. 2024 Oct 29;12(11):2180. doi: 10.3390/microorganisms12112180 (PMC11596864; doi:10.3390/microorganisms12112180)
Supplement: Supplementary file 1 [file microorganisms-12-02180-s001.zip › Miura_et_al_SupplementaryFigure.pdf]

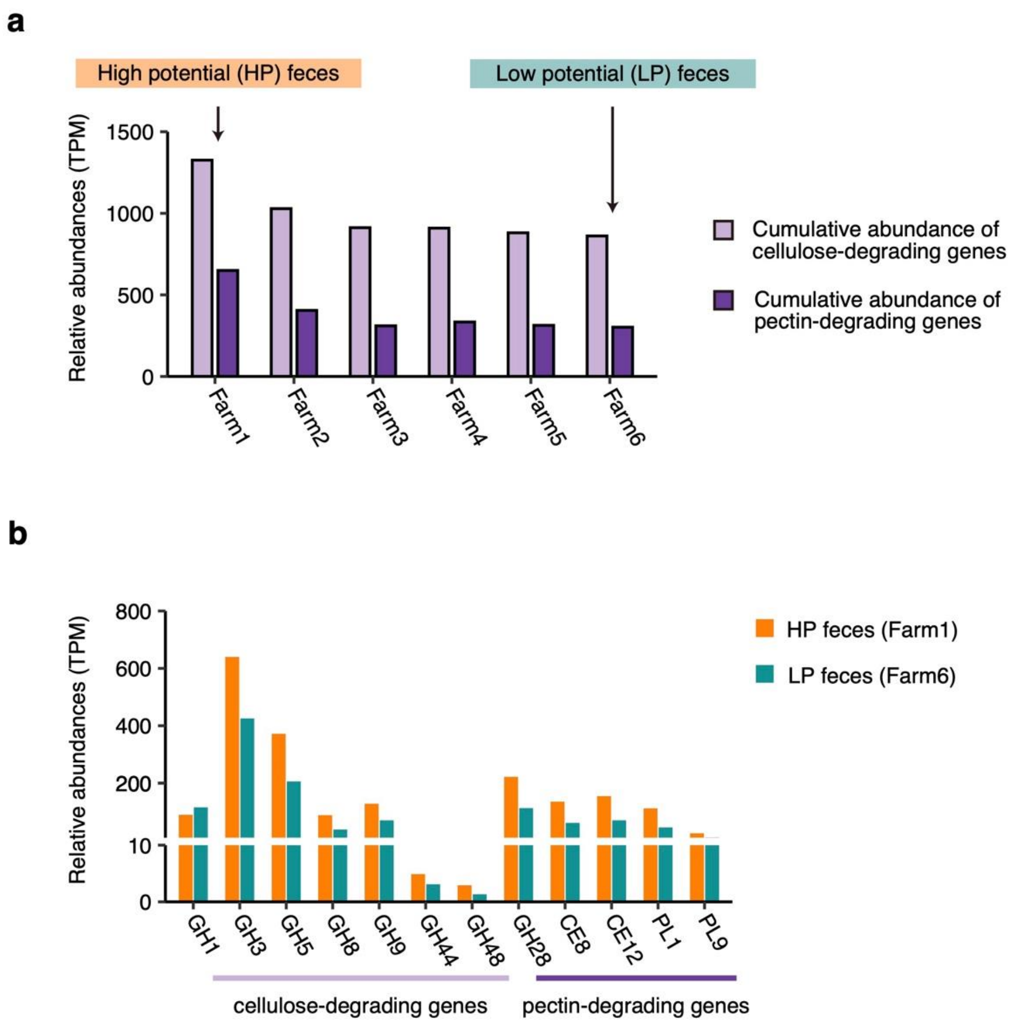


**Supplementary Figure S1. Evaluation of the fiber degrading potential of the sows' gut microbiota in six farms.** Feces were sampled from five sows (2nd–5th parity) from each of the six farms, which were not included in Experiment 1, and the samples from same farm were pooled at an equivalent volume. Pooled feces were subjected to microbial DNA extraction, according to Inoue et al. (2016), and to WG metagenomic library preparation and analysis in the same manner as for Experiment 1. **(a)** Cumulative relative abundances of cellulose- and pectin-degrading genes in the microbiota of pooled feces from six farms. Cellulose- and pectin-targeting CAZyme families were selected as per Flint et al. (2012) and are shown in Figure S1b. **(b)** Relative abundances of individual CAZyme family genes targeting cellulose and pectin in the feces with high (HP, Farm 1) and low (LP, Farm 6) potential for fiber degradation.

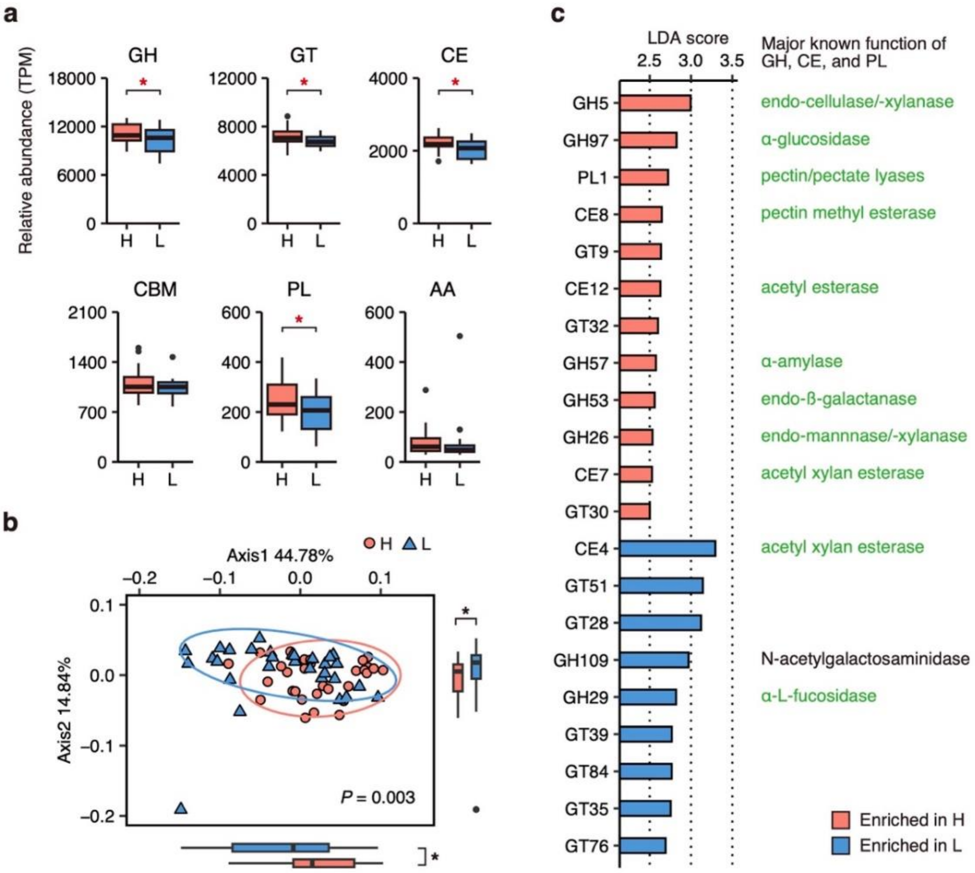


**Supplementary Figure S2. Comparison of the carbohydrate-active enzyme (CAZyme) gene profiles in the fecal microbiome of sows in groups H and L.** **(a)** Relative abundances of genes belonging to six CAZyme-classes, including glycoside hydrolases (GH), glycosyltransferases (GT), carbohydrate esterases (CE), carbohydrate-binding modules (CBM), polysaccharide lyases (PL) and auxiliary activities (AA) in groups H and L. Significance was tested using the Wilcoxon rank-sum test ($^{*}P < 0.05$). **(b)** Principal-coordinate analysis on Bray-Curtis dissimilarities based on the relative abundances of genes at the CAZyme family level. Individual symbols represent individual samples (red circle, sows H; blue triangle, sows L). Significance was tested using a permutational multivariate analysis of variance (PERMANOVA, permutation = 9,999). Boxplots show distributions of samples in groups H (red) and L (blue) along PC1 and PC2 ($^{*}P < 0.05$, Wilcoxon rank-sum test). **(c)** Differentially abundant CAZyme family genes in groups H and L. A linear discriminant analysis effect size (LEfSe) analysis was conducted, and only CAZyme families with significances (LDA score > 2.5, $P < 0.05$, FDR < 0.15) are shown. Captions next to the bars indicate major known functions of GH, CE, and PL (CAZy database, http://www.cazy.org/) and the green color indicates genes encoding plant-derived carbohydrates degrading enzymes.

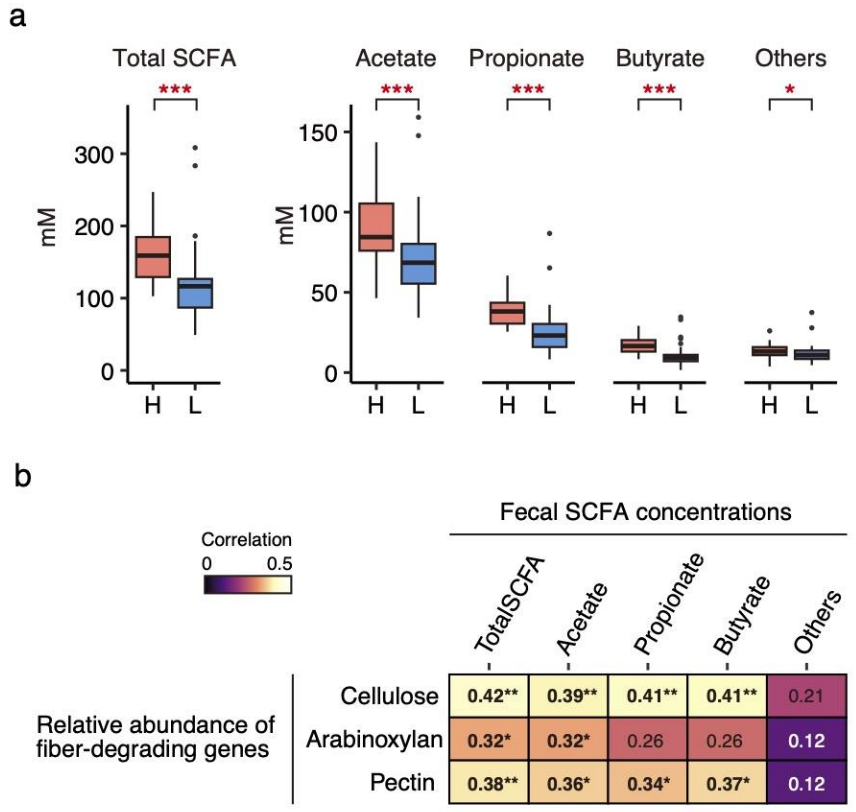


**Supplementary Figure S3. Fecal SCFA production and its relation to fecal microbial capacity for dietary fiber degradation in sows (a)** Comparison of fecal SCFA concentrations between groups H and L. Data of the SCFA concentrations of sows used in the present study were extracted from Uryu et al. [7] and reanalyzed. “Others” indicates the sum of the concentrations of valerate, iso-butyrate, and iso-valerate. Significance was tested using the Wilcoxon rank-sum test ($^{***}p < 0.001$, $^{*}p < 0.05$). **(b)** Spearman’s correlation between cumulative relative abundances (TPM) of cellulose-, arabinoxylan-, and pectin-degrading genes (Figure 2a) and fecal SCFA concentration (Supplementary Figure S2a). Values and colors indicate correlation values ($^{**}P < 0.01$, $^{*}P < 0.05$).

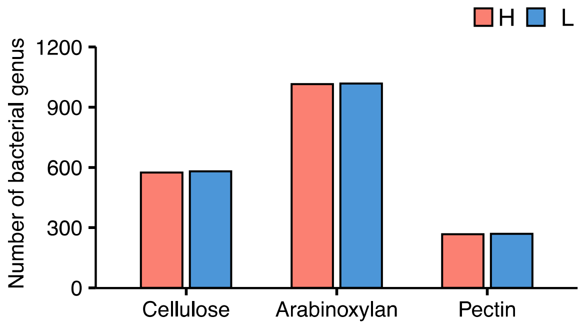


**Supplementary Figure S4. Total number of bacterial genera detected in affiliation of cellulose-, arabinoxylan-, and pectin-degrading genes.**
